# Cluster Headache: Diagnosis, Management, and Treatment in Pediatric Headache

**DOI:** 10.3390/jcm13051203

**Published:** 2024-02-20

**Authors:** Alessandro Borrelli, Massimiliano Valeriani, Gabriele Monte, Fabiana Ursitti, Martina Proietti Checchi, Samuela Tarantino, Giorgia Sforza, Laura Papetti

**Affiliations:** 1 Academic Department of Pediatrics, Bambino Gesù Children’s Hospital, Istituto di Ricovero e Cura a Carattere Scientifico (IRCCS), 00165 Rome, Italy; alessandro.borrelli@opbg.net; 2 Headache Center, Developmental Neurology Unit, Bambino Gesù Children’s Hospital, IRCCS, 00165 Rome, Italy; massimiliano.valeriani@opbg.net (M.V.); gabriele.monte@opbg.net (G.M.); fabiana.ursitti@opbg.net (F.U.); martina.proietti@opbg.net (M.P.C.); samuela.tarantino@opbg.net (S.T.); giorgia.sforza@opbg.net (G.S.); 3 Medicine Department, Hospital of Rome, Tor Vergata University, 00133 Rome, Italy; 4 Center for Sensory Motor Interaction, Aalborg University, DK-9220 Aalborg, Denmark

**Keywords:** cluster headache, pediatric, children, adolescents, headache, trigeminal autonomic cephalalgias, stabbing headache, indomethacin, migraine

## Abstract

Despite its rarity, cluster headache can affect children. Patients with cluster headaches often experience symptoms in their adolescence, but the time it takes for a correct diagnosis can be very long. Cluster headache can be mistaken for other pathologies, which can result in patients being diagnosed and treated incorrectly. CH therapy often represents a challenge in pediatric age as there are no studies dedicated to this age category and the therapy strategy is generally based on data from adult experience. The aim of this review is to provide a summary of the current literature on cluster headache in children and adolescents.

## 1. Introduction

Cluster headache (CH) is not common in pediatric age, but it can be diagnosed in headache centers for children and adolescents, which requires proper recognition and treatment. Undertreatment, significant disability, and pain are common in young patients, and even though CH has unique symptoms, the diagnosis is often delayed. Most pediatric cases begin in adolescence, after 15 years of age, but many cases have an onset at a younger age. CH differs from adulthood in terms of frequency of pain attacks and associated symptoms but maintains the same general characteristics [1]. Also, many cases (13–76%) show features of migraine, making a correct diagnosis a challenge for unexperienced physicians. Differentials encompass stabbing headache and other trigeminal autonomic cephalalgias (TACs), which may share some similarities with CH. Diagnosis is primarily based on the clinical picture, but neuroimaging is generally recommended to rule out other causes, mostly neoplastic [2,3]. This review is aimed at examining the available data for diagnosing, managing, and treating CH in developmental age.

## 2. Methodology

Here, we review the literature on CH in pediatric age. Pubmed and Scopus were used to search for articles on pediatric CH that have been published to date. The keywords we used are cluster, pediatric, children, adolescents, and trigeminal autonomic cephalalgias. Only articles that were available in English were included. We considered all types of articles, including case series, case reports, reviews, and meta-analyses, that reported on patients with an onset before 18 years of age.

Our search resulted in the identification of 20 articles that were published from 1994 to the present. 

We retrieved and included in our analysis 11 case-series or case-reports, 4 narrative reviews, 2 original articles, and 3 systematic reviews.

## 3. Epidemiology

The prevalence of CH in children is estimated to be between 0.03% and 0.1% [1,2,3], slightly less than the prevalence in adults, which is around 0.1–0.4% [4,5]. An online survey conducted in the USA on 1134 patients showed that around 35% of patients have their first episode before 20 years of age [6] while another study, also conducted in the USA with a similar methodology, reported that 27.5% of participants had an onset before 18. According to other data, only 5% of cases start before age 14, while 18% begin between age 15 and 19 [7]. Cases have been reported in all age groups, with patients having an onset as early as 1 year old. Females seem to have a slightly earlier onset age [8]. Around 10% of pediatric cases have a positive family history of CH [9].

The majority of cases occur between 15 and 25 years of age, with prevalence gradually diminishing for older age groups [9].

CH is more prevalent in male children and adolescents, similarly to what was observed in the adult population. A survey reported around 72% in pediatric age are male [6], but this male predominance might not be the case before puberty, as suggested by some published data that show a similar proportion between sexes under 10 years of age [9] and under 13 years of age [7]. Recently some authors published a case-series with a strong female predominance (male-to-female ratio of 1:1.7), but this might be due to selection bias [10]. Also, the abovementioned online survey shows that the relative majority of female patients (45%) have the first CH episode before the age of 20 [6]. These data, taken together, support the thesis that hormonal factors may have an important role and that estrogens may have a protective function, as other authors pointed out [11].

Among other environmental factors associated with CH, a greater risk has been described in children exposed to smoking during childhood [12,13,14]. 

## 4. Pathogenesis

The understanding of the pathophysiology of CH is still incomplete. Activation of trigeminovascular and parasympathetic systems is probably involved in CH and explains many of the characteristics of this disorder, such as the autonomic symptoms, response to vasoconstrictive agents like sumatriptan, and potential response to sphenopalatine ganglion (SPG) stimulations [2]. Furthermore, increased levels of calcitonin gene-related peptide (CGRP) and vasoactive intestinal peptide (VIP) support the role of these systems but do not justify the rhythmic and behavioral features of bouts [3,4]. Involvement of the hypothalamus and of the brain networks that process nociceptive information has been proposed [4]. It has been postulated that activation of the trigeminovascular system may be the cause of pain attacks, but what ultimately causes CH may be the underlying changes in the hypothalamus and in the pain-processing brain networks [2,3,4]. Dysfunction of the hypothalamus is also postulated on the basis of anatomical, neuroimaging, hormonal, and genetic studies [4]. In patients with CH, structural and functional changes in the hypothalamus and in brain areas involved in the pain modulator system have been described. Furthermore, neurophysiological studies showed altered pain perception and decreased pain thresholds in CH patients [4].

## 5. Diagnosis

Criteria for diagnosis of CH are described in the International Criteria of Headache Disorders 3rd edition (ICHD-3) [15].

At least five attacks fulfilling criteria B–D:

A. Severe or very severe unilateral orbital, supraorbital and/or temporal pain lasting 15–180 min (when untreated).
B. Either or both of the following:
  *a.*  at least one of the following symptoms or signs, ipsilateral to the headache:
    *i.*  conjunctival injection and/or lacrimation
    *ii.*  nasal congestion and/or rhinorrhea
    *iii.*  eyelid oedema
    *iv.*  forehead and facial sweating
    *v.*  miosis and/or ptosis
  *b.*  a sense of restlessness or agitation.
C. Occurring with a frequency between one every other day and eight per day.
D. Not better accounted for by another ICHD-3 diagnosis.

Pain is often very severe and often accompanied by restlessness, and for this reason CH is often nicknamed as “the suicide headache”. Episodic CH is defined by pain bouts lasting from seven days to one year, separated by at least three months of remission. Chronic CH is defined by pain bouts lasting more than 1 year without remission, or with a pain-free period lasting less than 3 months. If all but one of the criteria A-D are met, cases can be diagnosed as probable CH. The majority of cases in the pediatric literature are episodic, but some authors have reported high rates of chronic CH cases (18–23%) [7,10,16]. Females [7,9] and patients who are resistant to first-line treatment [9] seem to show a higher percentage of chronic CH, both in adult and in pediatric age. Circannual periodicity in bouts is quite frequent, with clusters often occurring in the same month of the year. Circadian rhythmicity is found in most pediatric cases, which present at the same time of day, and 36–75% of attacks happen at night or when waking up [10,17,18,19,20]. 

The ICHD 3 criteria were based on experience in the adult population, but their validity is widely accepted in the pediatric population as well. Despite this, there are some differences between childhood and adulthood [2]. As compared to adults, the frequency of attacks is lower (1–6 times per day) in children and adolescents [1,8]. Also, cranial autonomic symptoms and restlessness are less frequent than in adulthood, but nausea (13–76%), emesis (19–36%), photophobia (34–68%), and phonophobia (13–56%) are more common than in adulthood [7]. The last features may lead to misdiagnosis of CH as migraine [7,8]. As well as in other pediatric headaches, clinical evaluation must be based mainly on observation of the child’s behavior [1].

It is possible for CH diagnosis in pediatric age to take longer than expected. A recent meta-review found a gap of around 2 years and 4 months between first clinical signs and diagnosis [17], while a recent case-series reported a mean delay in diagnosis of 1.9 years (1 month–7 years) [10]. The differential diagnoses of CH are secondary causes that can mimic CH as well as other primary headaches. Although a diagnosis of CH must be made on a clinical basis, given the low frequency in pediatric age, neuroimaging is routinely recommended at the time of diagnosis to exclude conditions that could mimic this disorder. The study must be preferably a brain magnetic resonance imaging (MRI) with vascular study in order to exclude secondary causes, primarily neoplasms in the pituitary and cavernous regions and vascular lesions [3,4,21,22,23].

It seems that symptomatic cases in pediatric age may often be associated with a diagnosis of probable CH (as defined in ICHD-3) rather than fulfilling all the criteria [10,24,25]. Eberhard et al. described five cases of symptomatic CH, but only one case of hypothalamic pilocytic astrocytoma with carotid stenosis presented all ICHD-3 criteria, the others being diagnosed respectively with Graves’ disease, atypical optic neuritis, prolactinoma, and congenital right eye blindness ipsilateral to the attacks [10]. Yu et al. reported another case that met all the criteria for a CH diagnosis after cardiac stent placement [24]. 

The decision to perform neuroimaging in all adult patients with CH, even with typical symptoms, is being discussed. According to some authors, a neuroimaging examination should only be carried out if there are atypical features in the clinical history or neurological examination [22,26]. An onset in childhood, considering the low frequency, should however be addressed as a red flag and demands neuroimaging [2,3,5].

Differential diagnosis with other primary headaches can be a challenge in pediatric age. Migraine without aura is one of the most difficult conditions to distinguish from CH. This is of great importance because patients with CH do not respond to migraine treatments. The duration of migraine attacks in children can be as short as 4 h, which is one of the main confounders. ICHD 3 criteria have established a minimum duration of 2 h for migraine attacks in children [15]. This means that CH and migraine attacks can overlap in duration. Another fact to consider is that 70% of the cases of migraine show cranial autonomic features [27,28], complicating the distinction between the two. In CH, pain is typically unilateral and often associated with restlessness. On the other hand, migraine patients usually lie down and prefer to remain still in a quiet environment [8,21]. Furthermore, autonomic signs in migraine are generally bilateral rather than unilateral, and in most cases, they include conjunctival injection and lacrimation [29]. In CH, autonomic signs are commonly unilateral to the site of pain and, in the paediatric population, they most frequently include lacrimation (81%), conjunctival injection (61%), nasal congestion (57%) [8], ptosis (52%), rhinorrhea (45%), facial sweating (16%), miosis (14%), and eyelid edema (3%). Erythema of the eyelid has also been reported in two cases with probable CH [30,31].

Stabbing headache (SH) also has some characteristics that may be confused with CH, primarily when showing with an orbital and severe pain. SH is characterized by transient and localized stabs of pain in the head that occur spontaneously in the absence of organic disease of underlying structure. Each stab lasts for up to a few seconds and recur with irregular frequency, from one to many per day [15]. The most important distinction with CH is that there are no autonomic symptoms (Table 1). 

It may sometimes be difficult to distinguish CH from other TACs, particularly from paroxysmal hemicrania (PH). TACs share some features like pain that is monolateral and severe and the association with autonomic symptoms and/or restlessness. The differentiation between TACs must be based on different attacks’ duration, frequency, and response to indomethacin [15]. If we use the duration of the attack as a reference, on the two opposites we find short-lasting unilateral neuralgiform headache attacks with cranial autonomic symptoms (SUNA) and short-lasting unilateral neuralgiform headache with conjunctival injection and tearing (SUNCT), both showing with attacks lasting a few seconds and hemicrania continua (HC) in which the pain is non-remitting for at least 3 months. In these cases, the duration of the attacks allows a relatively easy diagnosis (Table 1). Also, SUNA/SUNCT are generally characterized by greater frequency of attacks (3 to 200 attacks per day) than CH. Differently, PH has overlapping characteristics with CH in terms of duration (2 to 30 min) and frequency of the attacks (more than five per day). It has been stated that differentiation between CH and PH can be aided by a trial with indomethacin, since CH is traditionally refractory while PH has been described as dramatically responsive. However, cases of SUNCT, SUNA, and CH with atypical features that were responsive to indomethacin were also described [2]. Also, some typical PH and HC were described that were unresponsive to indomethacin [30]. When we have a patient with a suspected TACs showing with clinical characteristics that do not allow a definitive diagnosis, it is always worth attempting treatment with indomethacin. In fact, some patients can benefit greatly from this therapy. However, it should be considered that there are TACs cases with undefined or overlapping characteristics [2,3,30]. Acknowledging this difficulty in the differential diagnosis should prompt clinicians to carefully reevaluate patients and reconsider alternative diagnosis when the treatment is not effective.

Other diagnoses that can mimic CH are trigeminal neuralgia and rhinosinusitis secondary headache (“headache attributed to disorder of the nose or paranasal sinuses” in ICHD 3). Trigeminal neuralgia is characterized by unilateral pain in the distribution of trigeminal nerves and may be accompanied by mild autonomic symptoms such as lacrimation or redness of the ipsilateral eye. Distinction from CH may be guided by duration, which is lower than 2 min in the vast majority of cases, lack of a circadian and circannual rhythmicity, and precipitation by innocuous stimuli in the affected trigeminal distribution [15]. Trigeminal neuralgia in pediatric age is an extremely rare condition, and the few cases described in the literature have secondary etiology mainly due to vascular pathologies [3,8,10].

Headache caused by rhinosinusitis is typically associated with other symptoms and/or clinical signs of this disorder. A diagnosis of this condition requires clinical, nasal, endoscopic, and/or imaging evidence of acute rhinosinusitis. Also, association of TACs to autonomic signs, such as tearing or nasal congestion, may lead to confusion between these two conditions. To distinguish them, it must be remembered that in CH the symptoms are strictly unilateral, have a paroxysmal progression, and the pain is very disabling [15]. It should be kept in mind that many cases of CH are initially confused with rhinosinusitis and treated inadequately [14]. 

## 6. Therapy

Medications that are used in the pediatric age have been borrowed from the experience in the adult population. Furthermore, the efficacy measures in pediatric age are sometimes based on studies that also include patients who do not meet all the CH criteria and who are better characterized as probable CH [2,17,31]. Therapies are generally divided into three stages: acute, preventive, or transitional. Unlike adults, in pediatric age, we have no guidelines or controlled data on efficacy and safety for the treatment of CH (Table 2 and Table 3) [22,32]. However, some authors have tried to give indications on the treatment of CH in pediatric age. These suggestions are based on efficacy data from studies on adults with CH and safety data from pediatric case series [21,33,34].

### 6.1. Acute Treatments

In adults, the best treatments to interrupt a CH attack are oxygen, subcutaneous sumatriptan, and triptans in nasal spray, which should be used according to guideline recommendations [22,32]. 

During pain attacks, the most effective abortive agent in most patients is inhalation of 100% oxygen at a dose of 7–12 L/min for 15 to 30 min through a non-breathing mask. The resolution of the attack after exposure to oxygen has been proposed as a quick test to support the diagnosis of cluster headache. Furthermore, oxygen therapy has no adverse effects [21,22,23]. Although subcutaneous sumatriptan is effective in adulthood, it was rarely used in the pediatric setting and resulted in unsatisfactory outcomes: out of five cases published, it showed good efficacy only in two [10,31]. Subcutaneous sumatriptan is also available at doses of 3 mg/0.5 mL and 4 mg/0.5 mL. Although in the acute treatment of CH subcutaneous sumatriptan was effective at a dosage of 6 mg/0.5 mL [10,31], lower doses could be considered in low-weight children or when side effects cannot be tolerated.

In children between 12 and 17 years old, sumatriptan nasal spray has been proven to be well-tolerated, and in children 6–10 years old, it has been demonstrated to be well-tolerated at 5 mg dose. Intranasal zolmitriptan is approved for migraine patients 12 years of age or older and has been reported to be effective in pediatric CH [10,16]. In a meta-review, both intranasal triptans were found to be effective in more than 75% of cases [17].

To ensure that pediatric patients with CH receive appropriate treatment and avoid ineffective medications, it is important to disclose and explain the use of oxygen and triptans in the healthcare setting. Most of the retrospective case-series in literature show the use of ineffective or unrecommended drugs. In a recent meta-analysis conducted by Bastos et al., they reported that 51 patients with CH were treated with oxygen or triptans in approximately 51% of cases, and the response rates were over 66%. The majority of patients had previously been given NSAIDs and acetaminophen, with a response rate of approximately 20% [17]. Eberhard et al. reported that in 25 cases of CH, the oxygen inhalation was used only in one case, intranasal zolmitriptan in two cases, and intranasal sumatriptan in six cases [10]. Instead, rizatriptan and oral sumatriptan, unauthorized in the pediatric age and not recommended in CH, were used in a total of 12 cases [10].

### 6.2. Preventive Treatments

The spectrum of proposed preventive treatments is wider than that of abortive treatments, with an even smaller number of pediatric reported cases for most of the options. As for the treatment of the attack, the level of evidence and recommendations in pediatric CH is suboptimal [17]. Verapamil is the first choice as a preventive therapy in adults, followed by lithium or topiramate, and high doses of melatonin are the third choice [22,35,36]. Verapamil, topiramate, and melatonin have been strongly recommended for pediatric use [21].

Verapamil is usually administered at a typical target dose of 3–10 mg/kg/day, which can be gradually reached by monitoring the PR interval (the time from the onset of the P wave to the start of the QRS complex) on an electrocardiogram. Side effects include hypotension, fatigue, bradycardia, and atrioventricular (AV) block. Verapamil was effective in every pediatric case included in the meta-review by Bastos et al. [17]. Also, a recent retrospective case series confirmed that it is the most effective preventive treatment for children with CH [10]. 

Topiramate was effective in four out of six cases included in Bastos’ meta-review [17]. Recently published case-series by Eberhard [10] found efficacy in two out of nine cases included, but their data are limited by the inclusion of secondary, probable-CH, and CH-like cases, which may have lower responsiveness. The target dose for topiramate in pediatric age is 1–2 mg/kg/day divided into two doses. Adverse effects may include decreased serum bicarbonate, hyperammonemia, dizziness, weight loss, abdominal pain, and nausea.

Lithium is a second-line treatment for adults [22], and it requires regular checks of lithium hematological levels. Side effects include tremor, acne, hypothyroidism, and lithium prescription in children is limited. We found only three cases in which it was used [16,20], and it was considered effective only in one case of a 13-year-old male with chronic CH.

Valproic acid or sodium valproate was used in four cases and showed at least partial efficacy in three cases, with sustained good efficacy only in one and transitory good efficacy in another. Its use in female patients is limited due to its teratogenicity. Tremors, fatigue, and weight gain are some of the other side effects.

Melatonin, given at a pediatric dose of 0.1–0.2 mg/kg/day, has been proposed as a valid treatment for children with CH given its good tolerability. However, efficacy in pediatric CH was reported only in one case [20]. Side effects include sleepiness and dizziness [22,32].

Usage of drugs that are not part of the adult guidelines has been described in pediatric CH by some authors [10,16,17,20,24].

Gabapentin was effective in six out of seven cases in which it was used [10,16,24,25]. Out of the seven cases, three were reported as symptomatic but fulfilled all the criteria required for a diagnosis of CH. Furthermore, gabapentin proved to be effective in all primary cases. Usual pediatric doses range from 8 to 35 mg/kg/day divided in three doses, but gabapentin must be initiated with 5 mg/kg in a single dose and adding one dose every day until the third day of therapy. Ataxia, dizziness, drowsiness, and fatigue are some of the common adverse effects [24,25].

Indomethacin was traditionally considered ineffective in CH, but it has been proven effective in some reported cases of CH [10,14,16,18,19,20,34,35,36]. Its use can either be acute or preventive, but many studies in the literature do not report which type of administration was used [16,20,36]. Treatment with indomethacin in pediatric age starts at a dosage of 1 mg/kg/day and is increased every 2–3 days until symptoms are controlled (maximum 225 mg/day). In order to prevent gastrointestinal effects, it is better to split the daily dose into three administrations [37].

Propranolol was used in a total of five pediatric cases of CH or probable CH [10,16,19,38] and was considered effective in three of them. The dose of 0.5–3 mg/kg/day is administered to children in divided doses, and its negative effects include sleep disorders in infants, bronchitis, bronchospasm, and hypotension [19,38].

Methysergide, an ergot derivative, was effective in three out of four pediatric cases [16,20]. However, this drug has been restricted by the EMA, and it has been withdrawn from the American market by the Food and Drug Administration (FDA) due to concerns that it could cause retroperitoneal fibrosis [39].

Pizotifen was effective in six out of nine cases in which it was used, but it is only available in a few countries and it is not approved by the FDA or European Medicines Agency (EMA) [16,38,40].

Greater occipital nerve (GON) block injections were successfully used with a combination of bupivacaine and triamcinolone as an acute treatment, followed by monthly nerve blocks with anesthetics in a 16-year-old female with chronic cluster headache [10].

Galcanezumab, a monoclonal antibody to calcitonin gene-related peptide (CGRP), showed efficacy as a preventive treatment in adults with episodic CH (and not in chronic CH) and has therefore been licensed by the FDA for this indication in adults [22]. To our knowledge, no studies reported administration of galcanezumab in pediatric CH, but phase 3 clinical trials are in progress to test its efficacy in pediatric migraine. Given its efficacy in adults, galcanezumab and other CGRP- related drugs may offer an interesting option for studies in the future.

Based on available data, verapamil must be considered the first-line prophylactic treatment in pediatric-age CH, similarly to adult CH [21]. Other options must be considered in patients with contraindications or in cases of poor tolerance to verapamil. Possible alternatives are gabapentin, topiramate, melatonin, and valproate, while scheduled GON injection might offer another interesting treatment option.

### 6.3. Transitional Treatment

Transitional or bridge therapies are started along with preventive medications, while waiting for the effectiveness of the latter, which may take days or weeks of high-dose treatments [22].

In adults, corticosteroids are effective transitional medications that can be used for short periods, and they have been suggested in children [2,3]. A group of 11 CH patients were treated with oral prednisone at a dose of 2 mg/kg/day for 17 to 20 days, and 100% efficacy was achieved within 5 days [14]. Arruda et al. showed that oral prednisone can be effective within a few days at a dosage range of 20 to 40 mg/day [20]. Another report of a 7-year-old female treated with intravenous prednisone for 15 days showed efficacy [34]. In a review of 19 pediatric CH, corticosteroid therapy was efficacious in 17 cases [36].

GON injection with lidocaine (2%) and depo-methylprednisolone was recommended as a transitional treatment in adults [22] and, given its good safe profile and tolerability, it has also been proposed in pediatric age. However, efficacy of GON block has not been studied satisfactorily in this age group [21,41]. Puledda et al. reported that unilateral injection with 30 mg of 1% lidocaine and 40 mg of methylprednisolone was effective in reducing the intensity and frequency of the attacks in three pediatric subjects with CH [42]. Non-invasive vagus nerve (nVNS) and sphenopalatine ganglion (SPG) stimulations have been recommended in episodic CH, but they have not been studied in the pediatric population [22].

### 6.4. Lifestyle Interventions

Exposure to tobacco has been associated with attacks’ severity and with the risk of transition from episodic to chronic CH [4; 13]. An association to tobacco exposure was reported also in pediatric CH [7; 14]. Smoking cessation in the household is advisable for many reasons; however, a survey conducted on adult CH patients by Ferrari et al. stated that most patients who quit smoking do not report benefits in terms of length, intensity, and maximum number of attacks per day [43,44].

Alcohol consumption can elicit attacks in many patients during the symptomatic period, leading some of them to spontaneously avoid its use. Also, some studies reported higher use of recreational drugs in patients than in the general population, often as self-medications during the attacks [45,46]. Educational therapy regarding alcohol, tobacco, and substance abuse may be indicated mainly in adolescents.

## 7. Limitations

Our review of the literature is limited by the exclusion of studies that are not available in the English language. Also, pediatric literature is scarce, and to our knowledge, none of the therapies that were recommended in pediatric CH were validated in a clinical trial. Data regarding efficacy of therapies are based mainly on case series, case reports, and retrospective studies, and as stated in the body of this review, they are often based on inclusion of both primary and secondary forms of CH, with some of the reported cases not meeting all the criteria for a definitive CH diagnosis.

## 8. Conclusions and Future Directions

Although many of the treatments available in adulthood are not authorized or well validated in pediatric age, some efficacious therapies exist and must be used. CH recognition and correct management is a challenge in pediatric neurology, given its severity and the burden it can cause on patients. One hundred percent oxygen inhalation and intranasal sumatriptan are first-line abortive treatments. The preferred option for prevention of CH attacks is verapamil, but melatonin, gabapentin, topiramate, valproic acid, and indomethacin may be evaluated. Waiting for preventive treatments’ efficacy, oral corticosteroids are effective, but also GON injections may be used, given their tolerability.

Future studies will hopefully circumstantiate better the efficacy of available treatments.

## Figures and Tables

**Table 1:** Common characteristics and differences of TACs, stubbing headache, and migraine without aura.

	Cluster Headache	Migraine	Paroxysmal Hemicrania	Stabbing Headache	SUNCT-SUNA	Hemicrania Continua
Prevalence in childhood	0.03–0.1%	7.7–17.8% [1]	0.05%	3.35–5.1%	0.01%	Unknown
Quality of pain	Sharp, throbbing	Pulsating	Sharp, throbbing	Stabbing	Stabbing, burning	Pressing, stabbing
Intensity of pain	Severe	Moderate-severe	Severe	Variable	Moderate-severe	Moderate-severe exacerbations
Site of pain	Unilateral, orbital, temporal	Unilateral or bilateral, usually frontotemporal	Unilateral, orbital, temporal	Variable	Unilateral, orbital, temporal, other trigeminal distribution	Unilateral
Duration of the attacks	15–180 min	2–72 h	2–30 min	0–120 s	5–240 s	Continuous pain for >3 months
Attack frequency	0.5–8/day	Variable, usually lower than the others	>5/day	Irregular, variable often only 1/day	3–200/day	Continuous pain with exacerbations
Cranial autonomic features	yes	70% in pediatric age [2]	yes	no	yes	yes
Restlessness	yes	No	yes	no	Not required	yes
Indomethacin response	No	Yes	Yes	no	No	Yes
Sex ratio in childhood (M:F)	2.5–4.3:1	1:1 in children 1:2 in adolescents [3]	1:1	1:1.6	1.5:1	1:2
Acute Treatments	Oxygen (abortive) Zolmitriptan nasal spray (abortive) Verapamil	NSAIDs Triptans	Indomethacin	Not applicable	Lamotrigine Topiramate Gabapentin	Indomethacin
Preventive Treatments	Verapamil Topiramate Melatonin	Calcium channel blockers Topiramate Amytriptiline Nutraceutics	Indomethacin Celecoxib Melatonin [4]	Indomethacin Carbamazepine Nutraceutics	Same as acute treatment	Indomethacin Celecoxib Melatonin [4]

Abbreviations: TACs–trigeminal autonomic cephalalgias; M–male; F–female; NSAIDs–non-steroidal anti-inflammatory drugs.

**Table 2:** Abortive treatment and recommendations for adult age.

Treatment	Dose	Side Effects	Evidence AAN 2016/Recommendations EAN 2023 (Adults)
Oxygen 100%	7–12 L/min over 15 min	NR	A/Strong
Intranasal solmitriptan	Pediatric: 2.5–5 mg Adults: 5–10 mg	paresthesia, chest tightness, nausea, asthenia, dizziness	A/Strong
Intranasal sumatriptan	Pediatric: 5–20 mg Adults: 20 mg	paresthesia, chest tightness, nausea, asthenia, dizziness	B/Strong
Subcutaneous sumatriptan	6 mg	site reactions, nausea and vomiting, dizziness, fatigue, paresthesia	A/Strong
Oral zolmitriptan	5–10 mg	paresthesia, heaviness, asthenia, nausea, dizziness, chest pain	B/Weak
Intranasal lidocaine	1 mL 4–10%	nasal congestion, unpleasant lidocaine taste	C/Consensus
Subcutaneous octreotide	100 ug	nausea, abdominal bloating, and diarrhea	C/Consensus
Vagus nerve stimulation	2 min	headache, dizziness	U/Strong Episodic
SPG stimulation	15 min	lead revisions, device explants loss of sensation, infection	B/Strong

Abbreviations: AAN–American Academy of Neurology; EAN–European Academy of Neurology; NR–not reported; SPC–sphenopalatine ganglion.

**Table 3:** Preventive treatment and recommendations for adult age.

Medication	Dosage	Side Effects	Evidencec AAN 2016/Recommendations EAN 2023 (Adults)
Oral verapamil	Pediatric: 3–10 mg/Kg/day Adults: 240–960 mg/day	hypotension, fatigue, constipation, edema, bradycardia, AV block	C/Consensus (first option)
Oral melatonin	Pediatric: 0.1–0.2 mg/Kg/day Adults: 10 mg/day	daytime sleepiness, headache dizziness, hypothermia	C/Consensus (third option)
Oral topiramate	Pediatric: 1–2 mg/kg/day Adults:25–400 mg/day	cognitive concerns, weight loss, renal stones, decreased sweating.	U/Consensus (second option)
Oral lithium	Adults: 800–900 mg/day	tremor, acne, hypothyroidism, muscle weakness	C/Consensus (second option)
Oral valproate	1000–1200 mg/day	tremors, fatigue, weight gain thinning hair, birth defects.	U/Consensus (third choice)
Intranasal civamide	100 uL of 0.025%	nasal burning, lacrimation, pharyngitis, rhinorrhea	B/weak episodic
Warfarin	Initiated at 2 mg/day (consider INR)	epistaxis, skin bruising	C/weak chronic
Vagus nerve stimulation	Three self-administered consecutive 2 min	headache, dizziness	U/Strong (add-on treatment) episodic
GON stimulation		shock-like sensations (kinking of wires)	U/Consensus (if all treatment have failed)
Subcutaneous galcanezumab	300 mg/month	local reaction, hypersensitivity, constipation	U/weak episodic

Abbreviations: AAN–American Academy of Neurology; EAN–European Academy of Neurology; AV–atrioventricular; INR–international normalized ratio; GON–great occipital nerve.
